# Association Study of the β2-Adrenergic Receptor Gene Polymorphisms and Hypertension in the Northern Han Chinese

**DOI:** 10.1371/journal.pone.0018590

**Published:** 2011-04-05

**Authors:** Yuqing Lou, Jielin Liu, Yao Li, Ya Liu, Zuoguang Wang, Kuo Liu, Hai Wu, Qiuli Niu, Wei Gu, Yanhong Guo, Zhizhong Li, Shaojun Wen

**Affiliations:** 1 Department of Hypertension Research, Beijing Anzhen Hospital, Capital Medical University and Beijing Institute of Heart Lung and Blood Vessel Diseases, Beijing, People's Republic of China; 2 State Key Laboratory of Genetic Engineering, Institute of Genetics, School of Life Sciences, Fudan University, Shanghai, People's Republic of China; 3 Department of Cardiology, Peking University Third Hospital, Beijing, People's Republic of China; 4 Emergency Center of Heart, Lung and Blood Vessel Diseases, Beijing Anzhen Hospital, Capital Medical University, Beijing, People's Republic of China; University of Texas M. D. Anderson Cancer Center, United States of America

## Abstract

**Background:**

The β2-adrenergic receptor (*ADRB2*) gene has been widely researched as a candidate gene for essential hypertension (EH), but no consensus has been reached in different ethnicities. The aim of the present study was to evaluate the possible association between the *ADRB2* gene polymorphisms and the EH risk in the Northern Han Chinese population.

**Methodology/Principal Findings:**

This study included 747 hypertensive subjects and 390 healthy volunteers as control subjects in the Northern Han Chinese. Genotyping was performed to identify the C-47T, A46G and C79G polymorphisms of the *ADRB2* gene. G allelic frequency of A46G polymorphism was significantly higher in hypertensive subjects (P = 0.011, OR = 1.287, 95%CI [1.059–1.565]) than that in controls. Significant association could also be found in dominant genetic model (GG+AG vs. AA, P = 0.006, OR = 1.497, 95%CI [1.121–1.998]), in homozygote comparison (GG vs. AA, P = 0.025, OR = 1.568, 95%CI [1.059–2.322]), and in additive genetic model (GG vs. AG vs. AA, P = 0.012, OR = 1.282, 95%CI [1.056–1.555]). Subgroup analyses performed by gender suggested that this association could be found in male, but not in female. Stratification analyses by obesity showed that A46G polymorphism was related to the prevalence of hypertension in the obese population (GG vs. AG vs. AA, P<0.001, OR = 1.645, 95%CI [1.258–2.151]). Significant interaction was found between A46G genotypes and body mass index on EH risk. No significant association could be found between C-47T or C79G polymorphism and EH risk. Linkage disequilibrium was detected between the C-47T, A46G and C79G polymorphisms. Haplotype analyses observed that the T-47-A46-C79 haplotype was a protective haplotype for EH, while the T-47-G46-C79 haplotype increased the risk.

**Conclusions/Significances:**

We revealed that the *ADRB2* A46G polymorphism might increase the risk for EH in the Northern Han Chinese population.

## Introduction

Essential hypertension (EH) is a worldwide escalating problem. In China, it was reported that 27.2% of the adult population age 35 to 74 years suffered from it [1]. EH is a highly heterogeneous disorder, which points to a multi-factorial aetiology and polygenic abnormalities [2]. As a consequence, a lot of gene polymorphisms have been assessed as candidate determinants of the risk of hypertension. In the search for the inheritable determinants of EH phenotype in humans, the gene encoding for the β2-adrenergic receptor (*ADRB2*) has been investigated worldwide, since in hypertension vascular responses to *ADRB2* stimulation are impaired [3], [4] and the *ADRB2* gene polymorphisms appear to affect vasodilation [5], [6].

At the molecular level, the role of the *ADRB2* gene in hypertension has been extensively evaluated. In vitro, the research [7] on the single-nucleotide polymorphisms (SNPs) in the coding region suggested that Arg16→Gly (rs1042713, A46G), Gln27→Glu (rs1042714, C79G), and Arg16→Gly +Gln27→Glu, compared to wild-type *ADRB2*, displayed normal agonist binding and functional coupling to the stimulatory form of G protein (G_s_), resulting in the stimulation of adenylyl cyclase activity. In vivo, it was reported that both polymorphisms might contribute to enhanced vascular reactivity to isoproterenol in capacitance vessel, which played a part in the regulation of blood pressure [5]. Another SNP at nucleotide position -47, C-47T (Arg-19Cys, rs1042711), is functionally important since it is located within a short open reading frame, the 5′-leader cistron, and affects *ADRB2* expression at a translational level [8]. Studies have also identified that C-47T polymorphism was in linkage disequilibrium (LD) with the A46G and C79G polymorphisms in the coding region [9]–[11].

A substantial number of studies have previously investigated the association between the A46G, C79G and C−47T polymorphisms and EH risk. Several of these failed to identify any association [11]–[18]. Other studies have found significant associations [9], [19]–[24], but there is no consensus regarding which allele was associated with hypertension or related traits. It has reported that the GG46 genotype remarkably increased the risk for EH in Japanese [19]. Interestingly, another study on the East Asian population suggested that the GG46 genotype played a protective role for EH in the Yi minority of Chinese [9]. Also, in the Han Chinese population, consistent results have rarely been found for particular candidate SNPs [23]–[25], as the Han Chinese population was considered be intricately substructured, corresponding roughly to Northern Han, Central Han, and Southern Han, based on the populations of the geographic origins [26]. To clarify the effect of these 3 polymorphisms on the risk of hypertension in the Northern Han Chinese population, we conducted a case-control study in middle-aged and older humans.

## Results

### Characteristics of the participants

A total of 1,137 unrelated participated subjects comprising 747 hypertensive patients (479 men and 268 women; mean age  = 51.52; SD±9.46) and 390 normotensive control subjects (233 men and 157 women; mean age  = 51.02; SD±7.66) were recruited for the present study. The clinical and laboratory parameters of cases and controls were summarized in Table 1. Aside from blood pressure measurements, significant differences in body mass index (BMI), total chelesterol, high-density lipoprotein cholesterol, triglyceride, glucose, the ratio of drinkers were observed between the hypertensives and the normotensives.

**Table 1:** Characteristics of study participants.

	Hypertension	Normotension	
	(n = 747)	(n = 390)	P
gender, M/F	479/268	233/157	NS
age(years)	51.52±9.46	51.02±7.66	NS
SBP(mmHg)	153.41±19.65	114.33±11.07	<0.001
DBP(mmHg)	99.18±14.20	74.04±8.26	<0.001
BMI(kg/m^2^)	26.88±3.45	24.90±3.20	<0.001
TC(mmol/L)	5.56±2.98	5.13±1.07	<0.001
HDL-C(mmol/L)	1.26±0.62	1.44±1.17	0.006
LDL-C(mmol/L)	3.38±0.87	3.45±0.78	NS
TG(mmol/L)	2.11±1.36	1.69±1.05	<0.001
Glu(mmol/L)	5.38±0.61	5.08±0.59	<0.001
Cr(µmol/L)	78.97±18.70	77.67±14.68	NS
ALT(U/L)	25.62±13.19	24.48±12.44	NS
HR(bpm)	71.30±9.61	70.73±9.20	NS
Smokers(n)	193	102	NS
Drinkers(n)	223	53	<0.001

SBP, systolic blood pressure; DBP, diastolic blood pressure; BMI, body mass index; TC, total cholesterol; HDL-C, high-density lipoprotein cholesterol; LDL-C, low-density lipoprotein cholesterol; TG, triglyceride; Glu, glucose; Cr, creatinine; ALT, alanine aminotransferase; HR, heart rate.

Values are mean±SD.

NS indicates not significant.

### Association analyses

Among all the participants, 99.3% samples of C-47T polymorphism, 98.3% samples of A46G polymorphism and 97.7% samples of C79G polymorphism were successfully tested in the laboratory. No deviation from Hardy-Weinberg expectation was observed for C-47T, A46G or C79G polymorphism in either hypertensives or normotensives. The genotype frequencies for the 3 polymorphisms in *ADRB2* were shown in Table 2. Univariate analyses indicated that the A46G polymorphism was significantly associated with EH. The significantly higher prevalence of G allelic frequencies was observed in the hypertensives than in the normotensives.

**Table 2:** The frequencies of the β2-adrenergic receptor gene C-47T, A46G and C79G polymorphisms genotypes.

		Genotype (frequency,%)			P*	Allele (frequency,%)		P**
C-47T		TT	CT	CC		T allele	C allele	
	Case (Total)	571(77.2)	163(22.0)	6(0.8)		1305(88.2)	175(11.8)	
	Control (Total)	314(80.7)	68(17.5)	7(1.8)	0.358	696(89.5)	82(10.5)	0.361
	Case (Male)	371(78.3)	100(21.1)	3(0.6)		842(88.8)	106(11.2)	
	Control (Male)	187(80.6)	41(17.7)	4(1.7)	0.723	415(89.4)	49(10.6)	0.726
	Case (Female)	200(75.2)	63(23.7)	3(1.1)		463(87.0)	69(13.0)	
	Control (Female)	127(80.9)	27(17.2)	3(1.9)	0.289	281(89.5)	33(10.5)	0.288
A46G		AA	AG	GG		A allele	G allele	
	Case (Total)	208(28.3)	369(50.2)	158(21.5)		785(53.4)	685(46.6)	
	Control (Total)	143(37.3)	174(45.4)	66(17.2)	0.003	460(60.1)	306(39.9)	0.003
	Case (Male)	135(28.8)	239(51.0)	95(20.3)		509(54.3)	429(45.7)	
	Control (Male)	94(40.9)	98(42.6)	38(16.5)	0.006	286(62.2)	174(37.8)	0.005
	Case (Female)	73(27.4)	130(48.9)	63(23.7)		276(51.9)	256(48.1)	
	Control (Female)	49(32.0)	76(49.7)	28(18.3)	0.167	174(56.9)	132(43.1)	0.164
C79G		CC	CG	GG		C allele	G allele	
	Case (Total)	566(76.8)	164(22.3)	7(0.9)		1296(87.9)	178(12.1)	
	Control (Total)	301(80.5)	66(17.6)	7(1.9)	0.335	668(89.3)	80(10.7)	0.337
	Case (Male)	367(77.9)	100(21.2)	4(0.8)		834(88.5)	108(11.5)	
	Control (Male)	185(80.4)	41(17.8)	4(1.7)	0.649	411(89.3)	49(10.7)	0.650
	Case (Female)	199(74.8)	64(24.1)	3(1.1)		462(86.8)	70(13.2)	
	Control (Female)	116(80.6)	25(17.4)	3(2.1)	0.319	257(89.2)	31(10.8)	0.319

*P value of the comparison of the additive genetic model using the generalized linear model.

**P value of the comparison of allelic frequencies.

Logistic regression analysis was performed after adjusting for confounding risk variables, that is, gender, age, BMI, total cholesterol, high-density lipoprotein cholesterol, low-density lipoprotein cholesterol, serum triglyceride levels, plasma glucose level, smoking habits and drinking habits. For A46G polymorphism, we observed a significantly higher prevalence of G allelic frequencies (P = 0.011, OR = 1.287, 95%CI [1.059–1.565]) in the hypertensives than the normotensives, which suggests that G allele might be a risk factor for hypertension in the Northern Han Chinese. Significant association could also be found in dominant genetic model (GG+AG vs. AA, P = 0.006, OR = 1.497, 95%CI [1.121–1.998]), in homozygote comparison (GG vs. AA, P = 0.025, OR = 1.568, 95%CI [1.059–2.322]), and in additive genetic model (GG vs. AG vs. AA, P = 0.012, OR = 1.282, 95%CI [1.056–1.555]). No significant association could be found between C-47T or C79G polymorphism and EH risk. (Table 3) Subgroup analyses were performed by gender and showed that significant effect between A46G polymorphism and EH risk could be found in male, but not in the subgroup of female. The P-value for A46G genotype-gender interaction was 0.617, which suggested that no significant interaction was found. As for the C-47T or C79G polymorphism, no significant association was found in either subgroup. (Table 3)

**Table 3:** Odds ratios of additive genetic model comparison for each single-nucleotide polymorphism genotype associated with essential hypertension in the Northern Han Chinese population.

		Overall		Male*		Female*	
SNP	Contrast	OR (95% CI)	P	OR (95% CI)	P	OR (95% CI)	P
C-47T	CC vs. CT vs. TT	1.012(0.746–1.374)	0.940	0.910(0.611–1.355)	0.642	1.156(0.709–1.887)	0.560
A46G	GG vs. AG vs. AA	1.282(1.056–1.555)	0.012	1.332(1.037–1.709)	0.025	1.176(0.854–1.623)	0.319
C79G	GG vs. CG vs. CC	1.032(0.760–1.401)	0.841	0.956(0.645–1.418)	0.825	1.138(0.692–1.873)	0.611

ORs adjusted for gender, age, body mass index, total cholesterol, high-density lipoprotein cholesterol, low-density lipoprotein cholesterol, serum triglyceride levels, plasma glucose level, smoking habits and drinking habits. OR, odds ratio; CI, confidence interval; SNP, single-nucleotide polymorphism.

*ORs adjusted for gender was not performed in Male and Female.

P-value for the interaction between A46G genotype and gender on hypertension was 0.617.

### Interactive effect of obesity and the *ADRB2* gene polymorphisms on hypertension

To explore the interactive effect of obesity and the *ADRB2* gene polymorphisms on hypertension, we analyzed the relation between C-47T, A46G and C79G polymorphisms and hypertension by stratification analyses. All the participants were divided into the obese subgroup and the non-obese subgroup according to BMI. The case-control study was further performed in both subgroups. Table 4 shows the genotype distributions and allele frequencies in obese and non-obese. As shown in Table 5, A46G polymorphism was related to the prevalence of hypertension in the obese (GG vs. AG vs. AA, P<0.001, OR = 1.645, 95%CI [1.258–2.151]). The association could also be found in the subgroup of obese men (GG vs. AG vs. AA, P = 0.005, OR = 1.603, 95%CI [1.153–2.227]) and obese women (GG vs. AG vs. AA, P = 0.028, OR = 1.739, 95%CI [1.062–2.849]). Whereas in the non-obese subjects, no significant association could be found between A46G polymorphism and EH risk. The P-value for A46G genotype-BMI interaction was 0.010. This result indicated that significant interaction existed between A46G genotypes and obesity on hypertension. There was no difference in genotype distribution of C-47T or C79G polymorphism between the hypertensive and the normotensive, neither in the subgroup of the obese subjects, nor in the non-obese participants (P>0.05).

**Table 4:** The genotype distributions and allele frequencies of the β2-adrenergic receptor gene C-47T, A46G and C79G polymorphisms in obese and non-obese.

Polymorphism	Total (n = 1137)				Male (n = 712)				Female (n = 425)			
	Obese (n = 713)		Non-obese (n = 424)		Obese (n = 487)		Non-obese (n = 225)		Obese (n = 226)		Non-obese (n = 199)	
	Cases	Controls	Cases	Controls	Cases	Controls	Cases	Controls	Cases	Controls	Cases	Controls
	(n = 533)	(n = 180)	(n = 214)	(n = 210)	(n = 365)	(n = 122)	(n = 114)	(n = 111)	(n = 168)	(n = 58)	(n = 100)	(n = 99)
C-47T												
TT (n)	397	145	174	169	277	98	94	89	120	47	80	80
CT (n)	125	32	38	36	81	23	19	18	44	9	19	18
CC (n)	6	3	0	4	3	1	0	3	3	2	0	1
C allele frequency	0.130	0.106	0.090	0.105	0.120	0.102	0.084	0.109	0.150	0.112	0.096	0.101
A46G												
AA (n)	142	74	66	69	101	53	34	41	41	21	32	28
AG (n)	258	82	111	92	176	53	63	45	82	29	48	47
GG (n)	124	22	34	44	80	15	15	23	44	7	19	21
G allele frequency	0.483	0.354	0.424	0.439	0.471	0.343	0.415	0.417	0.509	0.377	0.434	0.464
C79G												
CC (n)	395	141	171	160	275	97	92	88	120	44	79	72
CG (n)	126	32	38	34	81	23	19	18	45	9	19	16
GG (n)	6	3	1	4	3	1	1	3	3	2	0	1
G allele frequency	0.131	0.108	0.095	0.106	0.121	0.103	0.094	0.110	0.152	0.118	0.097	0.101

**Table 5:** Stratified analyses of association between the genotypes and risk of essential hypertension in the obese and the non-obese participants.

			Total		Male*		Female*	
SNP	Population	Contrast	OR (95% CI)	P	OR (95% CI)	P	OR (95% CI)	P
C-47T	Obese	CC vs. CT vs. TT	1.300(0.861–1.961)	0.211	1.138(0.672–1.923)	0.631	1.727(0.850–3.509)	0.130
	Non-obese	CC vs. CT vs. TT	0.708(0.430–1.166)	0.174	0.634(0.322–1.247)	0.187	0.753(0.351–1.618)	0.467
A46G	Obese	GG vs. AG vs. AA	1.645(1.258–2.151)	<0.001	1.603(1.153–2.227)	0.005	1.739(1.062–2.849)	0.028
	Non-obese	GG vs. AG vs. AA	0.927(0.688–1.248)	0.616	0.942(0.618–1.433)	0.777	0.839(0.539–1.305)	0.436
C79G	Obese	GG vs. CG vs. CC	1.287(0.851–1.942)	0.232	1.147(0.678–1.942)	0.609	1.647(0.810–3.356)	0.169
	Non-obese	GG vs. CG vs. CC	0.745(0.457–1.214)	0.236	0.722(0.380–1.374)	0.320	0.745(0.341–1.626)	0.459

ORs adjusted for gender, age, body mass index, total cholesterol, high-density lipoprotein cholesterol, low-density lipoprotein cholesterol, serum triglyceride levels, plasma glucose level, smoking habits and drinking habits. OR, odds ratio; CI, confidence interval; SNP, single-nucleotide.

*ORs adjusted for gender was not performed in sub-group analyses of Male and Female.

P-value for the interaction between A46G genotype and body mass index on hypertension was 0.010.

### Haplotype analyses

**Figure 1:**
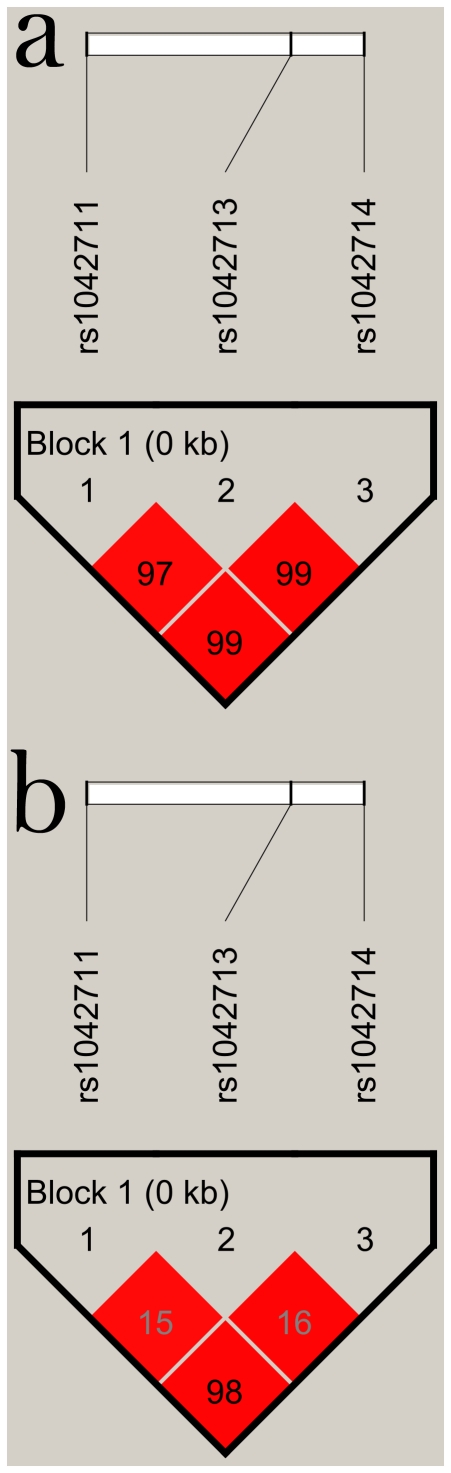
Linkage disequilibrium (LD) block defined by the Haploview program based on the confidence interval method. a represents LD measure of D'. b represents LD measure of r^2^.

As shown in Figure 1, the C-47T and C79G were almost in complete LD (D' = 0.99, r^2^ = 0.98). LD could also be found in C-47T and A46G (D' = 0.97, r^2^ = 0.15), as well as in A46G and C79G (D' = 0.99, r^2^ = 0.16). The Haploview program revealed C-47T, A46G and C79G polymorphisms in the same LD block. The haplotype analyses of the 3 polymorphisms of *ADRB2* in hypertension and control subjects were shown in Table 6. Only three of eight possible haplotypes (T-A-C, T-G-C and C-G-G) with frequency greater than 1% were detected in the haplotype analyses. Global analysis revealed that significant differences in haplotype distributions of *ADRB2* gene between cases and controls (*X* ^2^ = 8.49, df = 2, Global P = 0.014). Haplotype specific (HS) testing showed that the T-A-C haplotype was a protective haplotype (P = 0.003, OR = 0.763, 95%CI [0.639–0.912]) while the T-G-C haplotype was observed to be a risk (P = 0.015, OR = 1.265, 95%CI [1.047-1.530]). The C-G-G haplotype was not associated with EH in the Northern Han Chinese (P = 0.292).

**Table 6:** Haplotype analyses of the β2-adrenergic receptor gene polymorphisms in hypertension and control subjects.

			Haplotype frequency						
C-47T	A46G	C79G	Cases	Controls	HS test P value^a^	OR [95% CI]^a^	P value^b^	OR [95% CI]^b^	Global P value^c^
T	A	C	0.534	0.600	0.003	0.763 [0.639–0.912]	-	-	0.014
T	G	C	0.345	0.294	0.015	1.265 [1.047–1.530]	0.005	1.319 [1.085–1.604]	
C	G	G	0.121	0.106	0.292	1.162 [0.879–1.537]	0.089	1.284 [0.963–1.713]	

All haplotypes with frequency greater than 1% detected in the haplotype analyses are shown in the table.

HS test, haplotype specific testing; OR, odds ratio; CI, confidence interval.

a P values and OR values derived from comparing of a specific haplotype with the other two.

b P values and OR values derived from comparing each haplotype with the base-line haplotype (T-A-C).

c P value for global test comparing model with haplotypes to model without.

The relative effects of the haplotypes were evaluated by the logistic regression. As the most highly prevalent haplotype, the T-A-C haplotype was defined as the base-line haplotype. Comparing with the base-line haplotype, the T-G-C haplotype was associated with a risk to EH (P = 0.005, OR = 1.319, 95%CI [1.085-1.604]). No association could be found between the C-G-G haplotype and EH (P = 0.089).

## Discussion

The present study found that the A46G polymorphism of the *ADRB2* gene was associated with EH in the Northern Han Chinese. G46 allele had a significantly higher representation in the cases than in the controls. These findings were consistent with another study among the Northern Han Chinese on stage 2 hypertension diagnosed on systolic blood pressure (SBP)≥160 mmHg and/or diastolic blood pressure (DBP)≥100 mmHg reported by Gu *et al.* and Ge *et al*. [21], [23] Compared with their study, we failed to find significant association between C79G polymorphism and EH risk. As the diagnostic standard of Gu *et al.* and Ge *et al*.'s study [21], [23] was higher than our current study, we considered that C79G polymorphism might associate with more severe hypertension. Our previous meta-analysis [27] also found the similar results, which suggested that C79G polymorphism was associated with ‘severe hypertension' defined on SBP≥160 mmHg and/or DBP≥95 mmHg, but no significant association could be found based on the current clinical diagnostic standard of hypertension on SBP≥140 mmHg and/or DBP≥90 mmHg [2]. Interestingly, further research was conducted to compare with Gu *et al.* and Ge *et al*.'s study [21], [23] and different results were acquired. We also defined hypertensive participants according to the stage 2 definition and 441 patients were included. The patients' genotypes were compared with that of the normotensive subjects and logistic regression analysis was also performed after adjusting for confounding risk variables. There was no difference in genotype distribution of C79G between the cases and the controls (GG vs. CG vs. CC, P = 0.454, OR = 0.873, 95%CI [0.613–1.244]). We thought that this discrepancy might be resulted from 2 main reasons. Firstly, the participants of the stage 2 hypertension subgroup in the current study were fewer than that of Gu *et al.* and Ge *et al*.'s. For the low frequency of the uncommon allele of C79G, extremely large trial size was required for enough statistical power to detect real differences between groups[28]. The P-values of the case-control studies might have been a statistical fluctuation by chance owing to the nature of random sampling, dependent on the sample size and the low minor allele frequency of C79G. Secondly, different results might be partially attributed to the complex genetic differentiation among the Northern Han Chinese population[29]. We found almost complete LD between C79G and C-47T polymorphisms, whereas weak LD was found in Gu *et al.* and Ge *et al*.'s study. As for the C-47T polymorphism, both studies got negative results. Negative results were also found on C79G polymorphism in our case-control study since it was almost in complete LD with C-47T, which was different from Gu *et al.* and Ge *et al*.'s. Previous research also reported different degree of LD between C79G and C-47T in different ethnic background subjects[22], [30]–[32]. With the human migration in the history, the Northern Han Chinese population was under strong genetic influences from other populations[29]. The samples from our study and Gu *et al.* and Ge *et al*.'s were not entirely enrolled from the same area, the participants were not in a single homogenous population[26], [29], which might result in different association in these studies.

Stratification analyses showed that A46G polymorphism was association with EH risk in male, but not in the female population. No significant interaction existed between A46G genotype and gender. Yet we suggested that the different results between men and women and the P-value for genotype-gender interaction should be treated cautiously, as the women participants in this study were considerably smaller than men, which might limit the power to detect differences in OR estimates between the subgroups. Stratification analyses on obesity found the association between A46G polymorphism and EH risk in the obese subgroup, both in obese men and in obese women, whereas in the non-obese population, no association could be found. Significant interaction could also be found between A46G genotypes and BMI on EH risk. These findings suggested that obesity might have an effect on the association between genetic factors and EH risk. Mo *et al*. [25] compared the obese hypertensive population and the non-obese normotensive controls in the Southern Han Chinese population and indicated that the association could be found on C79G polymorphism in male, whereas no association was found on A46G polymorphism. We considered that the deviation between these 2 studies was mainly on the obesity effect. We performed stratification analyses and compared on the obese hypertension and the obese normotension, which was different from Mo *et al*.'s comparison on the control subjects. When both research studied on the same kind of participants, the non-obese population, and compared between hypertension and normotension, our findings were consistent with Mo *et al*.'s result. Furthermore, with the genetic background difference [26], [33]–[35] and the different environmental factors, such as eating habits, lifestyle, geographical localities and climate between the Northern and the Southern Han Chinese population, the results of these 2 studies might also be different.

Compared with the studies on Caucasians [11], [12], [15]–[17], most of them indicated ‘negative’ results on A46G polymorphism and EH risk. It was reported that the allele frequencies of the C-47T, A46G and C79G polymorphisms were quite different among ethnicities [36]. In the Asian populations, the allele frequencies of C-47, G46 and G79 were significantly lower than that of the Caucasians and the Blacks. The heterogeneity of allelic frequencies among the ethnicities might be the main point of the different results in the case-control studies. Beside this, the environment risk factors should also be taken into consideration. In the present study, A46G polymorphism was associated with EH risk in the obese population. This might be related to the living habits and environments of the obese. So we considered that the association might be the result of gene-environment interaction.

Haplotype analyses suggested that only 3 haplotypes with frequency greater than 1% were found in the study as previously described [30], [37]. As the haplotype frequencies listed in Table 6, the haplotype frequencies summed to 100%, both in cases and in controls, which meant that there was almost no rare haplotypes detected in the current study. This was attributed to the close LD of the C-47T, A46G and C79G polymorphisms. It was reported that subjects homozygous for G79 were nearly always homozygous G46, whereas A46/G79 occurred naturally extremely rare [38]–[40]. In our study, no AA46/GG79 combined genotype was found. As for the LD of C-47T and C79G, Lima *et al.* [30] reported that these two polymorphisms were in complete LD in whites and African Americans. In the current research, similar result was observed in Northern Han Chinese. In the 1105 subjects (97.2% of all the participants), whose genotypes of both the C-47T and C79G polymorphisms were successfully detected, only 4 subjects were deviated from the consistent genotype of mutant with mutant or wild-type with wild-type on these 2 tight LD polymorphisms. For the reasons of the high consistency of the C-47T and C79G polymorphisms, and of the low frequencies of both uncommon alleles, in the case-control study, the results of the haplotype analyses on the C-47T, A46G and C79G polymorphisms might be particularly determined by the allelic variants of the A46G polymorphism. Haplotype analyses showed that the T-A-C haplotype was a protective haplotype and the T-G-C haplotype was a risk, which was consistent with the findings of the association analyses between the A46G polymorphism and EH risk. For only 3 haplotypes detected, we could hardly perform further comparisons on multiple hypotype combinations.

As Green *et al.* reported [7], either the G46 allele or the G79 allele, compared to their wild allele, displayed normal agonist binding and functional coupling to Gs, resulting in the stimulation of adenylyl cyclase activity. In the downregulation of ADRB2, the A46G and the C79G polymorphisms played roles in the different degree. As compared to the wild-type AA46, the GG46 underwent a greater degree of downregulation of ADRB2 in response to the β2-agonist isoproterenol. These findings suggested that for the reason of decrease agonist sensitivity, an increased blood pressure might be produced by the GG46. On the contrary, the GG79, compared to the wild-type CC79, displayed no downregulation of ADRB2. Interestingly, the receptor with the GG46/GG79 combined genotype also displayed a greater degree of downregulation as compared to that found with the AA46/CC79 combined wild-type. Our results were in agreement with these findings. However, Wu *et al.* [9] studied on two Southern Chinese minority human population and got the different conclusions. They found that both the A46G and the C79G polymorphisms could play a role in the blood pressure regulation in the Yi, not in the Hani, minority group. In the Yi minority, the G46 allele and the G79 allele were more frequent in the control groups than in the hypertensive participants. Compared to Han Chinese, Yi and Hani minority groups have different ethnic origins [41], and living in a particular geographical environment. So we considered that the different results were probably due to the differences in ethnicity and geographical conditions.

It was reported [42] that the C-47T variant in the promoter region of the *ADRB2* gene controlled the *ADRB2* expression. The T-47 allele resulted in increased expression of the *ADRB2* as compared with the C-47 allele. As the LD between the C-47T polymorphism and the *ADRB2* gene coding block polymorphisms A46G and C79G, it suggested that the allele frequency of the C-47T polymorphism associated with that of the A46G and C79G polymorphisms. In other words, the A46G and the C79G variants within the coding region were thought to affect the *ADRB2* gene expression. The current study found that the A46G polymorphism, but not the C79G or C-47T polymorphism, associated with the risk of EH in the Northern Han Chinese population. However, for the tight LD between multiple polymorphisms on the *ADRB2* gene, we believe that the A46G polymorphism should not be independently considered as the impact factor of the *ADRB2* gene on the pathogenesis of EH. Further research on the role of the multiple polymorphisms of different regions within the *ADRB2* gene in the pathophysiologic of EH are important to clarify the genetic mechanism of EH.

Compared with early studies, the sample size of the present study was moderate. However, for the minor allele frequencies of C-47T, A46G and C79G of the Asians were significantly lower than that of the Caucasians and the Blacks as previously reported [36], and the allele frequencies of C-47 and G79 were much lower than G46, the statistical power to detect a significant association on C-47T and C79G polymorphisms were limited. The current study found that the 3 polymorphisms were in tight LD, but in the association study of EH, the results were quite different, both in all the participants and in the subgroups analyses. With the limited statistical power, the conclusions on C-47T and C79G polymorphisms should be treated cautiously. Considering the vastly different allele frequencies of the *ADRB2* gene polymorphisms in different ethnicities and the low minor allele frequencies of C-47T and C79G polymorphisms, larger case-control studies stratified for different ethnicities should be performed to clarify the association between the *ADRB2* gene polymorphisms and EH risk in the future.

In conclusion, we found that the A46G polymorphism in the *ADRB2* gene significantly associated with EH risk among the Northern Han Chinese population. The G46 allele was a risk factor for EH. Subgroup analyses performed by gender suggested that this association could be found in male, but not in female. Stratification analyses by obesity showed that G46 allele was related to the prevalence of hypertension in the obese population. Significant interaction existed between A46G genotypes and BMI on EH. Haplotype analyses suggested that the T-47-A46-C79 haplotype was a protective haplotype for EH, while the T-47-G46-C79 haplotype increased the risk of EH. Furthermore, for the tight LD between the polymorphisms in the *ADRB2* gene, further research is warranted to elucidate the role of the multiple polymorphisms within the *ADRB2* gene in the mechanism of EH.

## Materials and Methods

### Ethics Statement

The study complies with the Declaration of Helsinki, the local ethics committee of Beijing Anzhen Hospital of the Capital University of Medical Sciences has approved the research protocol. Written informed consent was obtained from each participant.

### Subjects

All individuals were of Northern Han Chinese origin and from the Beijing area recruited range from June 2008 to June 2009 in this study. All the participants were ascertained and identified via Anzhen Hospital of the Capital University of Medical Sciences, Beijing, China (n = 859), and two examination centers at local health stations, Liuliqiao (n = 71) and Guozhuang (n = 207), in Beijing suburbs.

Blood pressure was measured by trained and certified observers according to a common protocol adapted from procedures recommended by European Society of Hypertension [2]. After sitting for 30 min in a quiet room, three measurements with a standardized mercury sphygmomanometer were performed at least 5 min intervals. One of the 4 bladders (standard, larger, smaller, pediatric) was chosen and all readings were obtained from the right arm. SBP and DBP were defined according to Korotkoff I and V. Heart rate was measured by pulse palpation 30 sec after the measurement of blood pressure. Hypertension was defined as the average SBP>140 mmHg and/or the average DBP>90 mmHg and/or self-reported current treatment for hypertension with antihypertensive medication. The control subjects had systolic and diastolic blood pressures <140 mmHg and <90 mmHg, respectively, and should never been treated for hypertension [2]. Subjects with secondary hypertension, primary renal disease, diabetes mellitus, hepatic disorders, cancer, endocrine diseases such as hyperthyroidism were excluded. Physical examination, a questionnaire and serum biochemical profile were administered to each of the participants. Information on smoking and drinking habits was obtained by interview. Smoker was defined as the cigarette consumer who has smoked ≥100 cigarettes, and drinker was defined as the alcohol consumer who drank ≥12 times during the year [21], [23]. Obese was defined as a BMI ≥25 kg/m^2^ according to the World Health Organization obesity guidelines on Asians [25], [43], [44].

### Genotyping

Blood was taken into EDTA-containing receptacles. Genomic DNA was extracted from peripheral blood according to standard phenol-chloroform methods. We genotyped SNPs using the TaqMan assay. The *ADRB2* SNP Taqman probes and primers were obtained from Applied Biosystems Assay-by-Design Service for SNP genotyping. The sample DNA was amplified by PCR following the recommendations of the manufacturer. Thermal cycling was done on a GeneAmp PCR System 9700 thermal cycler (Applied Biosystems, 850 Lincoln Centre Drive, Foster City, CA 94404 USA). Genotypes were differentiated by analyzing the fluorescence levels of PCR products using an ABI PRISM 7900HT Sequence Detector (Applied Biosystems). Genotyping was performed blindly to all other data.

### Statistical Analyses for Patient–Control Study

We used SPSS (Version 17.0; SPSS, Chicago, Illinois) for database management and statistical analyses. All comparisons between specific groups for continuous variables were made using a two-sample t-test. Allelic and genotypic frequencies were compared between the hypertensive cases and the normotensive controls by using the chi-square test. Genotype frequency of the subjects specified by different genetic models (additive, dominant, recessive and homozygote comparison) was analyzed by multivariate logistic regression adjusted for covariates. Analyses used two-tailed estimation of significance. The statistical significance was defined P<0.05.

The presence of Hardy–Weinberg equilibrium was tested by the chi-square test for goodness of fit based on a web program (http://ihg.gsf.de/cgi-bin/hw/hwa1.pl).

Construction of the LDmap and haplotype blocks within C-47T, A46G and C79G polymorphisms of the *ADRB2* gene was based on genotypes and utilized Haploview software (version 4.1) (http://www.broad.mit.edu/mpg/haploview/) [45]. The expectation maximization algorithm [46] was performed to estimate haplotype frequencies and to obtain the best haplotype configuration for each multi-locus genotype. All haplotypes with frequency greater than 1% in the combined case and control samples were examined. The chi-square test was conducted to compare the haplotype distributions between the hypertensives and the normotensives. HS testing was performed to compare a specific haplotype with the others. Assuming the highly prevalent haplotype as the base line, each of the other haplotype was also compared with the base-line haplotype using a logistic regression model. A global test statistic comparing the model with genetic data to the model without genetic data was also performed to check for the overall association of haplotypes with the disease outcome, using an online computer platform SHEsis (http://analysis.bio-x.cn/myAnalysis.php) [47], [48].
